# Dietary Diversification and Specialization in Neotropical Bats Facilitated by Early Molecular Evolution

**DOI:** 10.1093/molbev/msab028

**Published:** 2021-03-04

**Authors:** Joshua H T Potter, Kalina T J Davies, Laurel R Yohe, Miluska K R Sanchez, Edgardo M Rengifo, Monika Struebig, Kim Warren, Georgia Tsagkogeorga, Burton K Lim, Mario dos Reis, Liliana M Dávalos, Stephen J Rossiter

**Affiliations:** 1 School of Biological and Chemical Sciences, Queen Mary University of London, London, United Kingdom; 2 Department of Ecology and Evolution, Stony Brook University, Stony Brook, NY, USA; 3 Department of Earth and Planetary Science, Yale University, 210 Whitney Ave, New Haven, CT, USA; 4 Escuela Profesional de Ciencias Biológicas, Universidad Nacional de Piura, Piura, Peru; 5 Escola Superior de Agricultura ‘Luiz de Queiroz,’ Centro de Energia Nuclear na Agricultura, Universidade de São Paulo, Piracicaba, Brazil; 6 Centro de Investigación Biodiversidad Sostenible (BioS), Lima, Peru; 7 Department of Natural History, Royal Ontario Museum, Toronto, ON, Canada; 8 Consortium for Inter-Disciplinary Environmental Research, Stony Brook University, Stony Brook, NY, USA

**Keywords:** molecular adaptation, positive selection, dietary evolution, branch-site model

## Abstract

Dietary adaptation is a major feature of phenotypic and ecological diversification, yet the genetic basis of dietary shifts is poorly understood. Among mammals, Neotropical leaf-nosed bats (family Phyllostomidae) show unmatched diversity in diet; from a putative insectivorous ancestor, phyllostomids have radiated to specialize on diverse food sources including blood, nectar, and fruit. To assess whether dietary diversification in this group was accompanied by molecular adaptations for changing metabolic demands, we sequenced 89 transcriptomes across 58 species and combined these with published data to compare ∼13,000 protein coding genes across 66 species. We tested for positive selection on focal lineages, including those inferred to have undergone dietary shifts. Unexpectedly, we found a broad signature of positive selection in the ancestral phyllostomid branch, spanning genes implicated in the metabolism of all major macronutrients, yet few positively selected genes at the inferred switch to plantivory. Branches corresponding to blood- and nectar-based diets showed selection in loci underpinning nitrogenous waste excretion and glycolysis, respectively. Intriguingly, patterns of selection in metabolism genes were mirrored by those in loci implicated in craniofacial remodeling, a trait previously linked to phyllostomid dietary specialization. Finally, we show that the null model of the widely-used branch-site test is likely to be misspecified, with the implication that the test is too conservative and probably under-reports true cases of positive selection. Our findings point to a complex picture of adaptive radiation, in which the evolution of new dietary specializations has been facilitated by early adaptations combined with the generation of new genetic variation.

## Introduction

The need to obtain food for the production of energy is a major driver of phenotypic and behavioural adaptation (Brown et al. 2004; Luca et al. 2010). Shifts in diet may open new niches and habitats, potentially promoting subsequent species diversification and phenotypic evolution (Greene 1983; Toft 1995; Rüber and Adams 2001; Darst et al. 2005; Russell et al. 2009; Davies et al. 2020). Within mammals, for example, dietary adaptation has shaped the recent and ancient evolutionary histories of diverse groups, from horses to primates, including humans (Andrews and Martin 1991; Ungar and Kay 1995; Perry et al. 2007; Mihlbachler et al. 2011; Hardy et al. 2015). In undergoing dietary transitions, species may have to overcome challenges associated with the uptake, transport, and breakdown (catabolism) of new macronutrients as well as rewiring other aspects of metabolism for efficient energy storage and synthesis (anabolism) of necessary compounds.

Of all mammals, bats offer unique opportunities for studying molecular adaptations associated with diet. As a taxonomic order, they show unparalleled dietary diversity and a suite of physiological and sensory adaptations for the acquisition and processing of food (Dumont et al. 2012; Fenton 2013; Jones et al. 2013; McNab 2013; Santana and Cheung 2016). However, studies of molecular adaptation in metabolism-related genes in bats have been limited in scope, typically focusing on small numbers of genes or, more recently, larger gene sets but in fewer species (Gutiérrez-Guerrero et al. 2020; Wang et al. 2020). Several lines of evidence have linked dietary shifts to relaxation of selection, sometimes with loss-of-function mutations (Jebb and Hiller 2018; Sadier et al. 2018; Sharma et al. 2018). In Old World fruit bats (Pteropodidae), for example, the nuclear factor erythroid 2-like 2 gene, *Nrf2*, has undergone amino acid replacements thought to reduce function (Yin et al. 2016). This transcription factor regulates expression of antioxidants and thus may be of reduced necessity to frugivorous bats with diets rich in vitamin C and other reactive oxygen species protectants. Tyrosine aminotransferase, *Tat*, a gene involved in amino acid catabolism has also experienced relaxed selection in members of the Pteropodidae (Shen et al. 2014). Other signatures of loss of function are seen in both digestive enzymes (Jiao et al. 2019) and sensory genes (Davies et al. 2020), mirroring wider findings in mammals (Jiang et al. 2012).

One family of bats in particular, the Neotropical leaf-nosed bats (Phyllostomidae), present an exceptional mammalian model for understanding the contribution of molecular adaptations to dietary specializations and diversification. Numbering over 180 species, members of this group have radiated from a putative insectivorous ancestor into a wide range of feeding guilds, which include insectivory, sanguivory (blood), carnivory, frugivory, and nectarivory. Consequently, throughout the evolutionary history of the group, there have been multiple shifts to new nutritional sources (Rojas et al. 2013; Saldaña-Vázquez et al. 2013; Saldaña-Vázquez 2014), characterized by different dietary macronutrient profiles that are expected to present contrasting metabolic challenges. For example, frugivorous and nectarivorous species ingest substantially more carbohydrate than protein. Nectar-feeders, in particular, experience higher postprandial blood sugar levels than other wild mammals (Kelm et al. 2011), which must be cleared to avoid glucotoxicity. Conversely, the blood-feeding common vampire bat, *Desmodus rotundus*, survives on very little dietary carbohydrate while mitigating the complications of excess nitrogen intake from its highly proteinaceous diet (Mendoza et al. 2018).

Related to the evolution of new feeding strategies, phyllostomids exhibit extreme variance in skull shape that exceeds that of any other mammalian clade (Dumont et al. 2014; Rossoni et al. 2017). Drastic craniofacial evolution has involved both lengthening and shortening of the skull and significant variation in the size and shape of cranial muscle attachments, mandibles, teeth, and tongues (Monteiro and Nogueira 2011; Dumont et al. 2012; Santana et al. 2012). Multiple findings point to the adaptive significance of rostral shortening in frugivorous leaf-nosed bats (Dumont 2004; Nogueira et al. 2009) for increased bite force for dealing with tougher fruit (Santana et al. 2010; Dumont et al. 2012; Santana et al. 2012) and rostral lengthening in nectar feeders for liquid diets (Dumont et al. 2014). In addition to diversification in skull shape, striking soft-tissue facial rearrangement is also a hallmark of the Neotropcial leaf-nosed bats, most evident in the extreme spear- or sword-like nasal appendages that characterize some lineages (Arita 1990; Teeling et al. 2002; Fenton 2010).

Studies of dietary genes in phyllostomids to date have uncovered some intriguing molecular adaptations. For example, frugivorous phyllostomids show a loss of the mitochondrial targeting sequence in the gene *Agt*, reflecting a shift toward peroxisomal targeting of its enzyme product alanine-glyoxylate aminotransferase 1, a condition also seen in Old World fruit bats (Pteropodidae) (Liu et al. 2012). Similarly, glycogen synthase (GYS), encoded by *Gys1*, shows a single inferred convergent amino acid change in two pteropodid and one phyllostomid species, albeit without positive selection (Fang et al. 2014), and shared amino acid substitutions between Pteropodidae and Phyllostomidae have been reported in phosphoenolpyruvate carboxykinase 1, a rate-limiting enzyme in the metabolism of glycogen to glucose (Zhu et al. 2015). Such shared changes might stem from diet-linked metabolic requirements during fasting periods. More recently, genome-wide analyses of smaller sets of taxa have discovered parallel amino acid substitutions between frugivorous pteropodids and some plant-feeding phyllostomids in genes encoding proteins involved in sugar and fat metabolism (Gutiérrez-Guerrero et al. 2020; Wang et al. 2020).

To gain a more complete understanding of the extent to which dietary diversification in this remarkable clade has been facilitated by selection acting on genes underlying food acquisition and metabolism, here, we describe the first large-scale screen for positive selection across all major dietary guilds within the Phyllostomidae, including ancestral branches. We predicted that diversifying adaptation to contrasting diets would involve largely discrete cohorts of positively selected loci, with dietary transitions accompanied by molecular adaptation in genes and pathways required to meet new metabolic demands. Notably, a major group of phyllostomids has transitioned from a putative insect-dominated diet to one that is rich in fruit and nectar. Thus, we hypothesized that branches corresponding to no change in diet, i.e. retention of the ancestral state, would show the fewest molecular adaptations relating to metabolism, whereas the branch leading to plant-based diets would show positive selection in genes involved in carbohydrate metabolism. Finally, we hypothesized that the subsequent evolution of highly derived nectar- and blood-based diets in some phyllostomid lineages would be associated with further molecular adaptations for overcoming the specific challenges of these respective extreme specializations. For a control that can be directly compared with the ancestral phyllostomid branch, we also performed our analyses on the branch leading to the sister clade Mormoopidae, which comprises all insectivorous members and is thus assumed to have not experienced any dietary shifts in its evolutionary history.

## Results

**Fig. 1.**
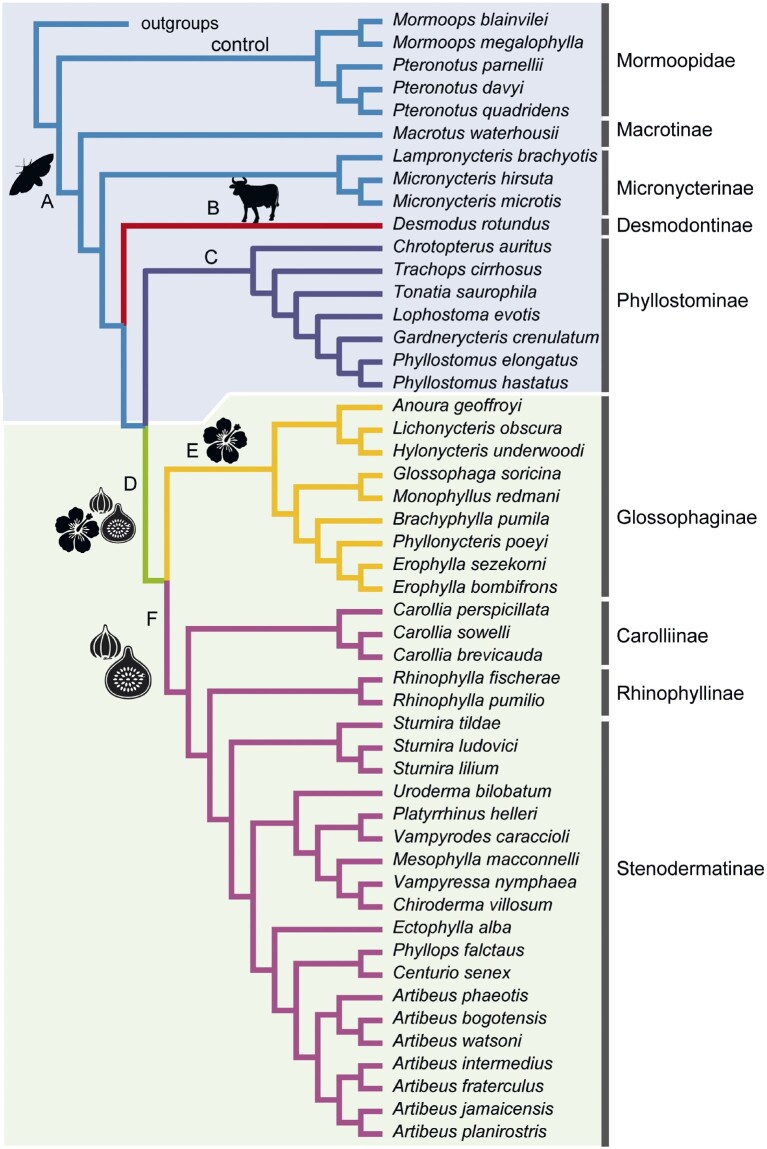
Cladogram of subfamilies in Phyllostomidae indicating branches tested for positive selection. (*A*) Phyllostomid ancestral branch, (*B*) *Desmodus rotundus*, (*C*) Phyllostominae ancestral branch, (*D*) plantivore ancestral branch, (*E*) Glossophaginae ancestral branch, and (*F*) frugivore ancestral branch. In addition, we tested the Mormoopidae branch as a control. The topology follows Rojas et al. (2016). Silhouettes indicate the diet of the inferred ancestor and all or most of the extant descendants from the branch: cow = blood-feeding, moth = insect-feeding, flower = nectar-feeding, fig = fruit-feeding. For details of outgroup species, see supplementary File 1, Table S1, Supplementary Material online.

We generated new RNA-Seq datasets for 58 bat taxa and assembled 89 de novo transcriptomes including two or more transcriptomes for some species (supplementary file 1, table S1, Supplementary Material online). We combined these with published coding gene sequences for 11 species (including *Homo sapiens* and additional bats) to produce multiple species alignments for 13,081 orthologous loci. The presence of mammalian BUSCOs in de novo transcriptomes varied, primarily depending on the original tissue sample (e.g. museum vs. flash-frozen in field), and whether multiple RNA-seq datasets from the same individual were combined into a single assembly (supplementary file 1, table S2, Supplementary Material online). For downstream analyses, we verified that at least two species from each ingroup clade of interest (Phyllostominae, Glossophaginae, frugivores) had a minimum percentage of complete BUSCOs of 70%, and that the common vampire bat, *Desmodus rotundus* had at least 72.1% complete BUSCOs, and that the control outgroup, Mormoopidae, two species had at least 56% complete BUSCOs. After quality control, each alignment contained 10–66 bat taxa and included representative sequences from all focal ingroup dietary guilds for analyses of molecular adaptation (fig. 1). The mean number of species per alignment was 45.0, the mean unmasked sequence length was 1,448.5 nucleotides, and the mean proportion of gaps was 5.9%. In total, 90.7% of alignments (*n* = 11,864) contained at least two members of each focal ingroup clade (Phyllostominae, Glossophaginae, and frugivores), and 69.6% (*n* = 9,102) contained at least two members of the control group Mormoopidae.

### Selection Tests

We performed branch-site tests of positive selection on six branches within the phyllostomid tree (branches *A*–*F*, fig. 1) and, as a control for branch A, the ancestral branch of the insectivorous sister family, Mormoopidae. Of the focal branches, dietary evolution along branches A and C (ancestors of Phyllostomidae and subfamily Phyllostominae, respectively) is thought to have involved no substantial shifts, given retention of the inferred ancestral arthropod-dominated diet by the most basal and/or the majority of descendent taxa, whereas B (common vampire bat, *Desmodus rotundus*) has involved extreme narrowing in dietary breadth to specialize on vertebrate blood. Branch D corresponds to a major transition from an animal- to a plant-based diet, whereas branch E (ancestor of subfamily Glossophaginae) involves subsequent narrowing in dietary breadth to specialize on nectar. Branch F (ancestor of a predominantly frugivorous clade) is inferred to represent no dramatic change, as frugivory is generally considered the less derived of plant-based diets and thus the likely ancestral state for plantivorous bats.

**Fig. 2.**
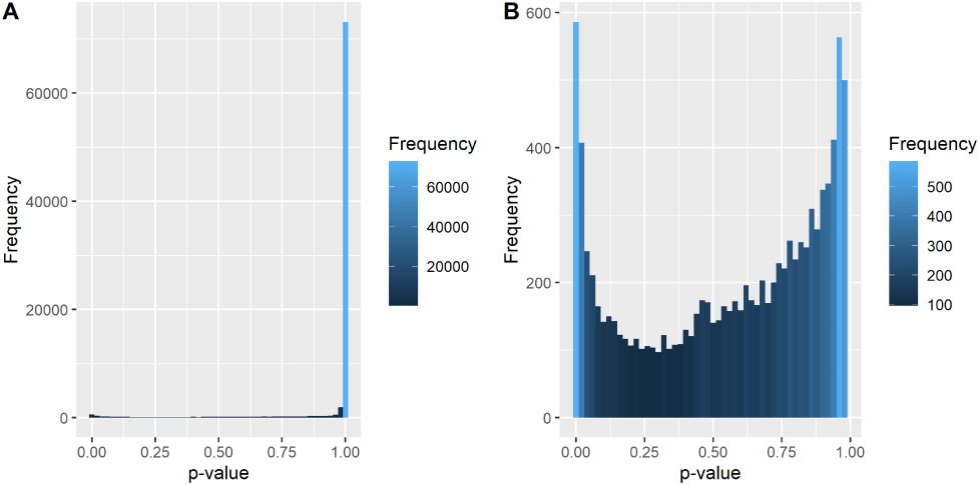
Empirical distribution of *P* values from branch-site tests. (*A*) *P* values from 84,951 selection tests, filtered for excessive BEB sites. 75.9% = 1. 87.8% > 0.98. (*B*) 10,369 *P* values < 0.98, filtered for excessive BEB sites.

We performed branch-site tests for positive selection across the seven focal branches, of which 1,330 cases (1.55%) were significant [*P* value < 0.05, likelihood ratio test (LRT)]. The number of retained significant results was reduced to 956 (1.11%) after filtering based on the distribution and prevalence of BEB Bayes empirical Bayes (BEB) sites. In 63,656 of the original tests (74.24%), the null model and alternative model returned equal log likelihoods (LRT statistic = 0, *P* value = 1). Furthermore, of the remaining 22,086 tests, over half had a null model log likelihood which was only marginally smaller than the alternative, such that the *P* value ≥ 0.98. Thus, only 10,899 (12.71%) of the original selection tests performed returned a *P* value < 0.98. It follows that our empirical distribution of *P* values was highly nonuniform, with an overwhelming majority at or very close to 1 (fig. 2).

### False Discovery Rate under Misspecification of the Null Model

Using a simple normal distribution example, performed simulations to examine the effect of model specification on LRT results. The first simulation reflected the case when the null hypothesis is always true [10,000 samples from *N*(0, 1)] and showed the correct *P* value distribution as a 50:50 mixture of χ^2^ and point probability mass at 1. Supplementary figure 2*A*, Supplementary Material online, shows the distribution of *P* values for the test, and supplementary figure 2*A'*, Supplementary Material online, shows the *P* value quantiles plotted against the quantiles from the uniform distribution. As expected, 50% of the *P* values have point mass 1 (these correspond to the 50% fraction of log-likelihood ratios equal to zero), whereas the remaining *P* values have the expected uniform distribution between 0 and 1 (after dividing by two, they would be distributed between 0 and 0.5). Using the false discovery rate (FDR) correction returned no positive tests at the 5% threshold.

We repeated this simulation but with 1,000 samples from the alternative model (i.e. with μ>0) and, as expected, found that for ∼10% of cases, the null model could be rejected. Supplementary figure 2*B*, Supplementary Material online, shows the distribution of *P* and supplementary figure 2*B'*, Supplementary Material online shows the corresponding quantile–quantile plot. Here, the proportion of *P* values at point mass 1 is 45% (half of 90%, or the proportion of times the null is true). An excess of *P* values close to zero is seen, corresponding to our true positives. Using the FDR correction at 5% (on the *P* values divided by two) gave 829 identified positives of which 34 are false positives. Thus, 34/829 = 4.1% which is close to the FDR threshold of 5%. If we do not halve the *P* values, not accounting for the 50:50 mixture due to the boundary condition, the test is much more conservative, returning fewer positives and fewer false positives: 15/789 = 1.9%.

Finally, we repeated the previous simulation with 4,500 samples from a misspecified null model (μ<0), 4,500 samples from the correct null model, and 1,000 from the alternative model. In this case, the proportion of *P* values at point mass 1 is too high at 67%, due to misspecification of the null model (supplementary fig. 2*C* and *C'*, Supplementary Material online). If we use an FDR correction at 5% and divide the *P* values by two, we still underestimate the proportion of false positives (2.3%, Table 1), which means that the test is too conservative. If we multiply the *P* values by the observed proportion of nonzero mass values (33%) and perform FDR, we detect more positives, but the observed proportion of false positives is still too low at 3.6% (Table 1). If we misspecify the null model 100% of the time in our simulations (not shown), the proportion of *P* values at point mass zero is 87.3%, and such an extreme case would further reduce our ability to discover true positives after FDR correction.

**Table 1.** Numbers of PSGs from branch-site tests for positive selection, including counts in functional categories of interest including seven reactomes and two GWAS-based categories (not mutually inclusive).

Branch	Genes tested	PSGs	PSGs (rFDR)	Dig.^a^	Vit.^b^	A.a.^c^	Lip.^d^	Ener.^e^	TCA^f^	Carb.^g^	Cran.^h^	Kid.^i^
Mormoopidae	9,091	89	21	2	1		4				5	5
Phyllostomidae	13,041	298	55	4		5	10	2	1	4	13	21
Desmodus	12,907	259	48	8	1	9	14		2	9	11	21
Phyllostominae	12,381	92	13	4	1		4	1	1		3	11
Plantivores	12,712	18	5					1			3	2
Glossophaginae	12,818	136	33	3		1	6			4	10	9
Frugivores	12,793	64	15	1	2		2	3		1	4	5

Note:—PSG, positively selected gene.

a Digestion and absorption.

b Metabolism of vitamins and cofactors.

c Metabolism of amino acids and derivatives.

d Metabolism of lipids.

e Integration of energy metabolism.

f The citric acid cycle and respiratory electron transport.

g Metabolism of carbohydrates.

h Craniofacial morphology (GWAS).

i Kidney function/disease (GWAS).

### Empirical SignificanceThresholds

Given that our empirical proportion of *P* values at or very close to 1 was ∼87%, which corresponded very closely to the simulation scenario in which the null model is misspecified 100% of the time, we could be certain that application of FDR to our *P* values would be too conservative. Focusing on *P* values < 0.98 produces a distribution closer to uniform, although still displaying a distinctive U shape, with an excess of *P* values close to 0 (a reliable indicator of true positives), and an increase in frequency as *p* becomes large (fig. 2). The persistent nonuniformity of *p*, even in the absence of the mass at 1, implied additional model misspecification beyond the nested boundary condition imposed by restrictions on ω.

Therefore, when the proportion of positively selected genes (PSGs) is small, as appears to be the case here, FDR correction is largely inappropriate and prevents detection of true positives. For this reason, we instead selected putative PSGs using the standard 5% threshold on unhalved LRT *P* values, with additional post-hoc filtering. We then closely examined annotations of metabolic, physiological, or morphological function in order to establish putative cases of molecular adaption linked to diet. To highlight PSGs with the greatest significance, we also applied a robust FDR (rFDR) correction to *P* values < 0.98 (Pounds and Cheng 2006), although it should be noted that the authors of this method explicitly caution against correcting nonuniform *P* values.

For all seven phylogenetic branches, we found that significant branch-site test results were not explained by the length or completeness of the gene alignment, implying that our results are real and not a consequence of data quality (linear model: gene LRT statistic ∼ number of taxa × sequence length).

### Numbers of PSGs

After filtering significant branch-site LRT results (*P *<* *0.05) based on BEB sites, we retained 298 putative PSGs for Branch A, 259 for Branch B, 92 for Branch C, 18 for Branch D, 136 for Branch E, 64 for Branch F, and 89 for the control branch (Table 1, supplementary file 2, Supplementary Material online). Therefore, we recorded the highest number of PSGs along the phyllostomid ancestral branch and the lowest along the plantivore ancestral branch. After application of rFDR to pooled *P* values < 0.98, numbers of PSGs were reduced, but consistent with the pre-rFDR findings (Table 1).

We cross-referenced the sets of PSGs for each branch with functional categories based on seven metabolic “Reactomes,” and, independently, with associations (GWAS) to selected phenotypic traits pertaining to either kidney function and excretion or craniofacial morphology (see Methods for details; see supplementary file 3 for a full categorization of PSGs, Supplementary Material online). For each branch, the cumulative number of PSGs belonging to any of the metabolic Reactomes generally mirrored the overall numbers of PSGs, although this proportion varied from 5.6% (D, plantivore ancestor: 1 PSG) to 15.4% (B, *Desmodus*: 40 PSGs). Thus, again contrary to expectations, the ancestral plantivore showed the lowest proportion of selection in loci with potential dietary significance, but, overall, there was no obvious variation between branches in the proportion of metabolic PSGs.

The membership of PSGs to particular Reactomes (e.g. carbohydrate metabolism) showed marked differences among the focal branches (Table 1), although numbers were generally too low (<5) to test for statistical differences (e.g. McGowen et al. 2020) (Table 1). For example, the branches leading to all phyllostomids (A) and to the common vampire bat (B) both showed positive selection in the carbohydrate metabolism Reactome (six and nine PSGs, respectively) despite the former’s inferred insectivorous diet being protein- and lipid-rich, and the latter’s diet overwhelmingly comprising protein. More consistent with our predictions, the insectivorous subfamily Phyllostominae and insectivorous control group Mormoopidae showed no apparent selection in the carbohydrate metabolism Reactome, whereas nectarivorous Glossophaginae had four carbohydrate PSGs (2.9% of its total PSGs).

Under GWAS-based categorization of PSGs, the proportion of selection in loci linked to kidney function/urinary excretion was relatively consistent across branches ranging from 5.6% for the control branch to 12.0% for C. Numbers were lower but proportions also similarly consistent across branches for links to craniofacial morphology: 3.3% (C)–7.3% (E). In both cases, the ancestral branch of plantivores (with the fewest total PSGs) had relatively high proportions of PSGs in these categories (11.1% and 16.7%, respectively), but these inflated proportions may be stochasticity, given the small numbers.

### Branch-Wise Findings

#### Phyllostomidae Ancestral Branch

**Fig. 3.**
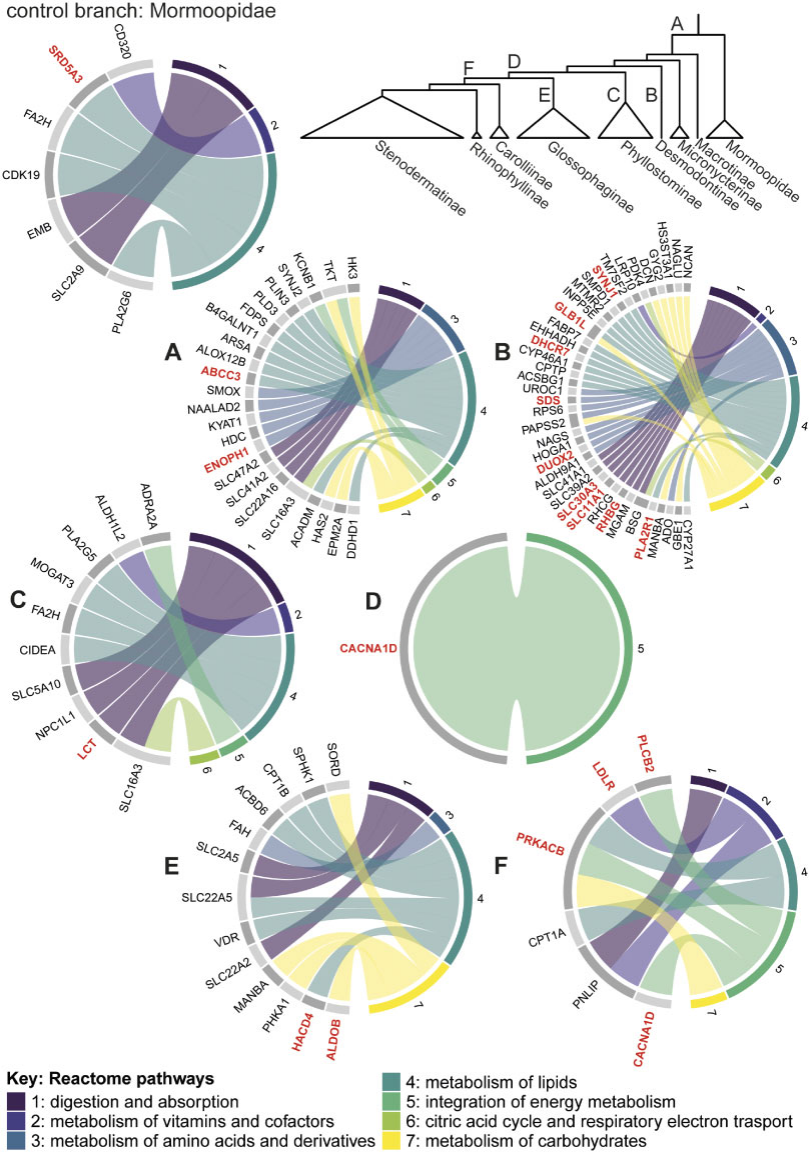
Functional roles of PSGs for each branch tested. (*A*) Phyllostomid ancestral branch, (*B*) *Desmodus rotundus*, (*C*) Phyllostominae ancestral branch, (*D*) plantivore ancestral branch, (*E*) Glossophaginae ancestral branch, and (*F*) frugivore ancestral branch. Up to seven Reactome categories are indicated by different numbers and colours on the right hemisphere of each chord diagram, see key for details. Gene names are given on the left hemispheres, and chord linkages indicate membership. Genes indicated in red remain significant after robust FDR correction. Only PSGs belonging to these categories are shown; for details of all PSGs, see supplementary files 2 and 3, Supplementary Material online.

The 298 putative PSGs detected at the base of phyllostomids included members of all major metabolic Reactomes, with five from the amino acid metabolism pathway, 10 from lipid metabolism and four from carbohydrate metabolism (fig. 3; see supplementary file 2 for PSGs with LRT statistics; supplementary file 3 for categorization, Supplementary Material online). PSGs from the amino acid Reactome included *ENOPH1*, involved in methionine biosynthesis, *KYAT1*, implicated in metabolism of the tryptophan metabolite kynurenine (Cooper 2004; Baumgartner et al. 2019), and *HDC*, which encodes the enzyme histidine decarboxylase, responsible for conversion of histidine to histamine, and is thus vital to numerous physiological processes such as immunity, neurotransmission, and digestion (Moya-Garcia et al. 2005). PSGs belonging to the lipid metabolism Reactome included *ACADM*, which encodes medium chain acyl-CoA dehydrogenase (MCAD), required for breakdown of medium chain fatty acids during beta-oxidation, the mitochondrial pathway for derivation of energy from lipids. Mutational variants cause MCAD-deficiency, characterized by symptoms of poor energy production such as hypoglycemia and lethargy, alongside the detrimental accumulation of medium chain fatty acids in tissues (Rinaldo et al. 1988). Additional PSGs in the lipid Reactome were *PLIN3*, a member of the perilipin family functioning in mobilization of fats in adipose tissue (Kimmel et al. 2010), the phospholipases *PLD3* and *DDHD1*, variants of which are linked to disorders of muscle strength and coordination (Tesson et al. 2012; Nibbeling et al. 2017), and *FDPS*, encoding an enzyme for production of farnesyl pyrophosphate, a vital intermediate in synthesis of sterols and carotenoids (Martín et al. 2007).

PSGs in the carbohydrate metabolism Reactome included *HAS2* and *EPM2A*. *HAS2* is responsible for synthesis of the ubiquitous matrix polysaccharide hyaluronan, important to cell and tissue architecture (Kosaki et al. 1999) and is also linked to facial morphology through GWAS. *EPM2A* specifies laforin, a protein believed to interact with another molecule, malin, to regulate unwanted glycogen deposition in tissues (Worby et al. 2008). Two further PSG members of the carbohydrate Reactome, *TKT* and *HK3*, have direct roles in the breakdown of dietary carbohydrate, encoding, respectively, transketolase, integral to the pentose phosphate pathway (Mitschke et al. 2010), and a cytoplasmic isoform of hexokinase, which plays a key first-stage role in most glucose metabolic pathways (Wyatt et al. 2010). Four further PSGs from the phyllostomid ancestral branch are solute carriers, belonging to the digestion and absorption Reactome, and include *SLC22A16* and *SLC16A3*. The former is a carnitine transporter with a role in the “carnitine shuttle,” the translocation of fatty acids to supply beta oxidation, whereas the latter is a proton-linked transporter of lactate and pyruvate and thus also of importance to the citric acid cycle (Fisel et al. 2013).

In addition to members of major metabolic Reactomes, numerous PSGs along the ancestral branch of Phyllostomidae are associated with either craniofacial morphology (13 genes) and/or excretion (21 loci) through GWAS. These include *ADAM28* and *CADM1*, both linked to cleft palate/orofacial clefting, and *NUF2*, *OSR1*, and *MAGI2*, associated with defined facial morphology factors. PSGs with possible importance to kidney function and waste processing include *RASGRP2*, *CYB5B* and *ANKRD33* (urate levels), *TRPA1* (gout), *PKD2* (many renal function traits), and *ANPEP* (gallstone disease). Of the latter two, *ANPEP* encodes a broad-specificity aminopeptidase expressed in membranes of the small intestine and renal microvilli (Bauvois and Dauzonne 2006), with a role in the digestion of peptides generated by gastric and pancreatic proteases, whereas *PKD2* is believed to function as a transmembrane cation channel in the kidneys, particularly for calcium (Qian et al. 1997). Calcium influx triggers multiple downstream pathways and reactions, and thus, expression of *PKD2* is likely to have a large role in kidney cell differentiation and function.

Two further PSGs of interest, specifically in the context of sugar metabolism, were *PPP1R3A* and *HKDC1*. *PPP1R3A* encodes the regulatory subunit of the heterodimeric enzyme glycogen protein phosphatase 1 (PP1) ( Delibegovic et al. 2003). By binding to muscle glycogen with high affinity, this subunit helps bring PP1 into proximity with other glycogen-associated enzymes such as GYS and glycogen phosphorylase kinase (PHK), which it can then dephosphorylate. Also important in glucose processing, *HKDC1* is a recently categorized hexokinase, believed to be a maternal-active isozyme, and is also implicated in diabetes (Kanthimathi et al. 2016).

#### Control Branch: Mormoopidae

On the ancestral branch of the phyllostomid sister clade, Mormoopidae, we detected putative positive selection in 89 loci. Within the major metabolic Reactomes, four PSGs belonged to lipid metabolism including the phospholipase *PLA2G6*, and *FA2H*, also under selection in Phyllostominae (fig. 3). Among other PSGs of interest in the context of diet, *SLC2A9* belongs to the digestion Reactome and encodes a solute carrier in the glucose transporter (GLUT) family with a specific role in the excretion and reabsorption of urate in the kidneys. Five PSGs were associated with craniofacial morphology through GWAS, including *SPON1* (facial morphology factor 1: breadth) and *GRN* (intracranial volume), whereas five could be linked to kidney function.

#### *Desmodus rotundus*: Sanguivory

We recorded 259 PSGs on the branch leading to sanguivory, with a strong signal pertaining to amino acid and lipid metabolism: 9 and 14 PSGs, respectively (fig. 3). PSGs belonging to the amino acid Reactome included *SDS*, encoding a serine dehydratase important to metabolism of serine and threonine (Yamada et al. 2008), *UROC1*, specifying a liver-localized enzyme mediating the second step of histidine breakdown (Espinos et al. 2009), and *HOGA1*, whose liver and kidney expressed product catalyzes the final step of hydroxyproline metabolism to glyoxylate and pyruvate (Monico et al. 2011). Another PSG important for protein metabolism, *NAGS*, encodes N-acetylglutamate synthase, which plays a central role in regulating the first step of the urea cycle (Schmidt et al. 2005). Among the 14 *Desmodus* PSGs from the lipid metabolism Reactome were *FABP7*, which plays a role in triglyceride metabolism and brain development (Arai et al. 2005), and *SMPD1*, a lysosomal acid sphingomyelinase that converts sphingomyelin to ceramide and thus may have an indirect role in cholesterol homeostasis (Simonaro et al. 2006). Genetic defects in *SMPD1* are associated with Niemann–Pick disease, in which harmful quantities of lipids accumulate in tissues (Ding et al. 2016). Also under apparent positive selection in this Reactome was *EHHADH*, involved in peroxisomal beta-oxidation of fatty acids (Houten et al. 2012), and *DHCR7*, responsible for the final stage of cholesterol synthesis.

We also detected selection in nine loci belonging to the carbohydrate metabolism Reactome, including *GYG2* and *GBE1*. *GYG2* encodes a glycogenin responsible for initial polymerization of glucose in the synthesis of glycogen, whereas *GBE1* encodes a late stage enzyme that catalyzes branching of glycogen side chains. Mutations in both loci can result in glycogen storage disorders (Mitchell et al. 2000; Imagawa et al. 2014). Positive selection was also detected in TCA-associated *PDK4*, a kinase which inactivates the key glycolytic enzyme pyruvate dehydrogenase.

We observed positive selection in a substantial number of PSGs linked to renal function and/or disease through GWAS: 21 loci in total. These include *ALDH16A1*, an aldehyde dehydrogenase associated with the glomerular filtration rate of creatinine and serum uric acid levels, and *NRXN2*, linked to urea and urate levels and renal overload. Other GWAS excretion PSGs include *FSTL4* and *FGFR2* (gout) and *ANKRD55* (urate levels). We also detected signatures of selection in 11 PSGs linked to craniofacial morphology through GWAS, including *CNTNAP2*, *RD3* and *BEST3* (facial morphology factors), and *ADAMTS8* (eye morphology).

#### Ancestral Branch of Phyllostominae: Insectivory and Other Forms of Animalivory

On the ancestral branch of Phyllostominae, 92 loci were identified as under positive selection, including four loci involved in lipid metabolism, four in digestion, one in the citric acid cycle, and one in vitamin metabolism, according to Reactome pathways (fig. 3). In the lipid metabolism Reactome, *CIDEA* has a putative role in thermogenesis and lipolysis (Barneda et al. 2015), *FA2H* is involved in sphingolipid production from fatty acids, whereas *MOGAT3* catalyzes diacylglycerol synthesis and possibly plays a role in dietary fat absorption in the small intestine. The four PSGs in the absorption/digestion Reactome included *NPC1L1*, *SLC5A10*, and *LCT*. *NPC1L1* encodes a membrane protein that mediates uptake of cholesterol by enterocytes in the intestine (Davis et al. 2004) and can also transport vitamin E. *SLC5A10* is thought to be a kidney-specific solute carrier, which functions as a sodium-dependent monosaccharide transporter (Wright and Turk 2004; Wright et al. 2007), whereas *LCT* encodes lactase, important in young mammals for digestion of milk. No PSGs were detected in the amino acid metabolism Reactome.

Other PSGs of putative significance to dietary specialization include three loci implicated in craniofacial morphology through GWAS, and 11 in kidney function, including SLC34A1, a sodium-phosphate cotransporter that has a major role in sodium influx in the kidneys (Iwaki et al. 2008). Additional loci of possible importance in a dietary context included *UGT2B4*, a bile-acid glucuronosyl transferase, which may have a role in conjugating, modifying, and eliminating toxins (Lévesque et al. 2010), and *PGA3*, which codes for a precursor of the gastric endopeptidase pepsin, crucial in digestion of dietary protein (Richter et al. 1998). Last, *SLC26A7* is a sodium-dependent anion transporter, which is predominantly expressed in the kidney (Xu et al. 2009). It permits exchange of bicarbonate, chloride, sulfate, and oxalate, thus playing a role in excretion and pH homeostasis.

#### Ancestral Branch of Plantivores

On the ancestral branch of the plantivorous clade, only 18 genes were found to be under putative positive selection. Of these, only one, *CACNA1D*, belonged to a Reactome pathway: the integration of energy metabolism (fig. 3). *CACNA1D* encodes a subunit of a calcium channel believed to be important for adrenal gland function and the production of aldosterone (Azizan et al. 2013).

#### Ancestral Branch of Glossophaginae: Nectarivory

On the ancestral branch of Glossophaginae, 136 PSGs were identified. Among these, PSGs for craniofacial morphology included *PRDM16*, previously showing selection in the phyllostomid ancestor. PSGs with roles urinary excretion included *SLC7A6*, a transporter of neutral and cationic amino acids (Hosoya et al. 2010) also implicated in glomerular filtration rate and metabolite ratios, and *SLC22A2*, an organic cation transporter (Jonker et al. 2003) encoding a kidney-specific protein that mediates flux of endogenous compounds and toxins. Multiple putative instances of molecular adaptation were found in lipid and carbohydrate metabolism pathways, with loci from both these Reactomes displaying signatures of positive selection (fig. 3). Among six lipid metabolism genes selected in Glossophaginae were *VDR*, *CPT1B*, and *SLC22A5*. *VDR* encodes the vitamin D receptor; fat-soluble vitamin D regulates the absorption of calcium and phosphate, integral to bone and tooth formation (Bouillon et al. 2008) and genetic defects in the receptor are linked to rickets. *CPT1B*, a member of the carnitine/choline acetyl transferase family, encodes a variant of carnitine palmitoyltransferase I, an enzyme critical to beta-oxidation of fatty acids (Miyagawa et al. 2008). Involved in the carnitine shuttle, it mediates transport of long-chain fatty acyl-CoAs from the cytoplasm into the mitochondria and, as such, is the rate-controlling enzyme in beta-oxidation. *SLC22A5*, also important to the carnitine shuttle, functions as a high-affinity sodium-dependent carnitine transmembrane transporter (Tamai 2013).

Four major loci belonging to the carbohydrate metabolism Reactome also displayed putative positive selection in Glossophaginae: *PHKA1*, *MANBA*, *ALDOB*, and *SORD*. *PHKA1* encodes the alpha subunit of PHK, a key enzyme in glycogen metabolism (Wuyts et al. 2005). β-Mannosidase, encoded by *MANBA*, is responsible for the lysosomal breakdown of mannose-containing polysaccharides. Mutational variants cause mannosidosis, the toxic accumulation of saccharides with neurodevelopmental symptoms (Riise Stensland et al. 2008). *ALDOB* encodes fructose-bisphosphate aldolase B, a key gluconeogenic/glycolytic enzyme responsible for reversible synthesis/breakdown of fructose-1,6-bisphosphate and has a central role in glycolysis (Santer et al. 2005). *SORD* encodes sorbitol dehydrogenasethat catalyzes the metabolism of the glucose alcohol sorbitol (Greene et al. 1987).

Additional PSGs in the ancestor of Glossophaginae with possible significance to dietary adaptation included the insulin-independent fructose transporter, *SLC2A5*, previously displaying a signature of positive selection on the ancestral branch of all phyllostomids, and *PPP1R3A*, the glycogen-associated protein phosphatase subunit highlighted by both the branch-site tests of the family and Phyllostominae ancestors.

#### Ancestral Branch of Frugivores

A total of 64 genes were found to be under putative positive selection in the ancestor of the frugivores. Two of these loci belonged to the lipid metabolism Reactome, including *CPT1A*, which encodes a subunit of carnitine palmitoyl transferase 1 (CPT1) similarly to *CPT1B* (fig. 3). Additional PSGs of interest included *LDLR* and *PNLIP*, both members of the vitamin/cofactor metabolism Reactome, and the latter also of digestion. *LDLR* encodes the low-density lipoprotein receptor, involved in cholesterol homeostasis, whereas *PNLIP* encodes pancreatic lipase. This enzyme is important for digestion of dietary fats through hydrolysis of triglycerides in the small intestine. Craniofacial PSGs included *GNB1L* and *ABCA4*, both associated with cleft lip, and *LINGO2*, linked to facial wrinkles.

### Functional Enrichment in Sets of PSGs

We also tested each set of PSGs from the seven branches for functional enrichment. In most branches, significantly enriched biological process (BP) gene ontology (GO) terms were diverse, including a substantial number of terms relating to immunity and cell signaling (supplementary file 4, Supplementary Material online). However, focusing on the most significant terms (*P *<* *0.01) revealed a degree of enrichment for functions related to dietary adaptation. On the phyllostomid ancestral branch (A), there was a strong signal for kidney function and water homeostasis, as GO : 0072208 “metanephric smooth muscle tissue development” was the top most significant term (*P *<* *0.001), with other terms including GO : 0003104 “positive regulation of glomerular filtration” and GO : 0045907 “positive regulation of vasoconstriction” among the most significant. Other highly significant terms for this branch included GO : 0030259 “lipid glycosylation” and GO : 0006508 “proteolysis.” For the control branch, Mormoopidae, the most significant term was GO : 0071404 “cellular response to low-density lipoprotein particle stimulus” (*P *<* *0.001), potentially reflecting adaptation in lipid metabolism, with other metabolic categories such as GO : 1903825 “organic acid transmembrane transport,” GO : 0090237 “regulation of arachidonic acid secretion,” GO : 0015820 “leucine transport,” GO : 0016095 “polyprenol catabolic process,” and GO : 0043605 “cellular amide catabolic process” among top terms. The most significant term for *Desmodus rotundus* (B) was GO : 0006885 “regulation of pH” (*P *<* *0.001), with other highly significant terms also pertaining to excretion, including GO : 0003148 “outflow tract septum morphogenesis,” GO : 0070634 “transepithelial ammonium transport,” and GO : 0070474 “positive regulation of uterine smooth muscle contraction.” PSGs for Phyllostominae (C) were enriched for GO : 0050995 “negative regulation of lipid catabolic process,” GO : 0046655 “folic acid metabolic process,” GO : 0009749 “response to glucose,” GO : 0015862 “uridine transport,” and GO : 0050892 “intestinal absorption,” whereas for Glossophaginae (E), GO : 0050917 “sensory perception of umami taste” was the third most significant term. For the ancestral branch of frugivores (F), GO : 0030299 “intestinal cholesterol absorption” was most significant (*P *<* *0.001), with GO : 1905167: “positive regulation of lysosomal protein catabolic process,” GO : 0006214: “thymidine catabolic process,” and GO : 0007202: “activation of phospholipase C activity” also among top terms. The ancestral branch of plantivores had the fewest metabolism-related enriched categories, but GO : 0001581 “detection of chemical stimulus involved in sensory perception of sour taste” was among the most significant.

## Discussion

Our analyses provide compelling evidence that dietary diversification among lineages of Neotropical leaf-nosed bats has involved pervasive positive selection in loci controlling metabolism and morphology at the base of this group, coupled with later signatures of selection coinciding with the evolution of highlyderived diets. In contrast with our predictions based on switches to novel diets, we found differences in the numbers and composition of PSGs across our focal lineages, with the greatest number detected on the ancestral branch of the family and fewest on the branch leading to the clade of plantivorous bats.

### Selection on Diverse Diet-Related Genes at the Base of Phyllostomidae

The ancestral phyllostomid bat, the ancestor of the sister clade Mormoopidae, and thus also their most recent common ancestor, have previously been widely considered to be insectivorous, suggesting little or no early dietary change. However, contrary to our expectations, we found that many of the genes showing positive selection along the ancestral phyllostomid branch encode proteins functioning in carbohydrate, protein, and lipid metabolic pathways as well as excretion processes with the former including key enzymes in the metabolism of dietary sugars (*TKT*, *HK3*, *HKDC*). Contrasting with this broad signal of metabolic adaptation in Phyllostomidae, we found proportionately more PSGs in the lipid metabolism and digestion/absorption Reactomes for the control branch, Mormoopidae, yet no apparent selection in the carbohydrate or amino acid metabolism Reactomes.

The presence of putative molecular adaptations for processing diverse macronutrients on the branch leading to phyllostomids implies that the ancestral form possessed a relatively generalized diet, supporting earlier speculations that relative morphological stasis across some phyllostomid lineages might be explained by a degree of frugivory in the ancestral taxon (Freeman 2000). Certainly, our results support the possibility that the genetic foundations for dietary diversification in this group may have been laid early in their evolutionary history, potentially facilitating later transitions to novel diets. For example, ancestral mutations in sugar metabolism genes may have functioned as a primer, allowing subsequent molecular evolution in additional carbohydrate metabolism loci to permit more specialized plant-based diets.

Similarly to the control branch, we recorded a relatively high proportion of PSGs associated with lipid metabolism and digestion/absorption for the phyllostomid subfamily Phyllostominae, also representing an insect-rich dietary specialization. Strong candidates for molecular adaptation include *NPC1L1*, implicated in lipid transport, with mutations linked to coronary heart disease and lipid storage disorder Niemann–Pick disease, and *PGA3*, which encodes a precursor of the major digestive protease pepsin. To date, there are no published studies of adaptations relating purely to insectivory in phyllostomids; however, duplications and selection in the lysozyme C gene have been related to chitin catabolism in members of the insectivorous bat family Vespertilionidae (Liu et al. 2014). Our results may thus reflect specific adaptations to the relatively higher protein and fat content of animal-based diets. Indeed, whereas most members of the subfamily Phyllostominae included in this study feed predominantly on arthropods, one focal species, *Trachops cirrhosis*, occasionally eats small frogs (Kalko et al. 1999), and unsampled larger members of this family are specialist carnivores (Vehrencamp et al. 1977).

### Limited Molecular Adaptation at the Origin of Plant-Based Diets

Perhaps, our most surprising result, contradicting our initial hypothesis, was an absence of clear diet-related molecular adaptations at the origin of plant-feeding. Of all branches tested, the ancestral branch of plantivores showed the fewest overall genes with signals of positive selection and only a single PSG within a metabolic Reactome: integration of energy metabolism. This finding further supports a scenario in which the molecular adaptations needed for processing the constituents of plant matter occurred long before the origin of an exclusive plant-based diet. The exploitation of pre-existing trait diversity, generated under different environmental conditions, has been proposed as an important mechanism allowing species to exploit new ecological opportunities during adaptive radiations (Gould 2002; Marques et al. 2019; Martin and Richards 2019). In the case of phyllostomid bats, the long lag between the establishment of substitutions in key genes and the emergence of novel diets could constitute a case of historical contingency, a phenomenon also recently proposed to explain the adaptive radiation of Antarctic fishes (Daane et al. 2019).

A similar but less extreme result was also obtained for the ancestor of the frugivorous bats, where PSGs included only a handful of metabolism-related genes alongside a slightly higher number of putative morphology-related genes. Certainly, the evolution of frugivory is considered one of the major ecological release points in the radiation of the Neotropical leaf-nosed bats, as a major burst of lineage diversification occurs after this point (Rojas et al. 2011; Rojas et al. 2012; Rojas et al. 2018). Indeed, the subfamily Stenodermatinae is often considered an adaptive radiation in its own right, showing unique selective signatures which differentiate it from the other frugivorous subfamilies Carolliinae and Rhinophyllinae (Dumont et al. 2012; Shi and Rabosky 2015; Rojas et al. 2016).

Our results may indicate that only a small degree of further adaptation was necessary to transition from an insect- to a plant-dominated diet. Establishing the nutritional profiles of insect and plant-based diets needs more work, however, several predominantly insectivorous species may supplement their diets with fruit (Uieda et al. 2007), whereas some plant-feeding phyllostomids ingest insects (Clare et al. 2014). Facultative fruit-insect omnivory may in fact be relatively common in Phyllostomidae, possibly resulting from ancestral adaptation to generalism at the base of the family, with subsequent exaptation to a fully plant-based diet (Freeman 2000). In such a scenario, early contact with plant-based foods would have precipitated metabolic adaptations necessary to cope with the higher proportion of sugar and water, and lower protein content, even before the emergence of cranial or dental adaptations for frugivory. Indeed, the idea that these bats might have been preadapted to undergo shifts in foraging and diet was recently proposed by Davies et al. (2020), who recorded a greater degree of positive selection in vision-related genes at the base of both the superfamily Noctilionoidea and in the ancestral phyllostomid, yet comparatively little adaptation at the inferred shift to plant-feeding.

### Extensive Positive Selection in Bats with Specialized Blood and Nectar Diets

The two most derived dietary specializations in phyllostomid bats are arguably blood- and nectar-feeding, each presenting unique metabolic challenges. The hematophagous diet of the vampire bat, unique among vertebrates, is, nutritionally, over 90% protein, placing considerable demands on excretory physiology (Breidenstein 1982; Schondube et al. 2001; Mendoza et al. 2018). Reflecting this, we detected selection in genes underlying amino acid metabolism and the clearance of toxic nitrogenous waste products as well genes with roles in kidney function and excretion, so mirroring this species’ physiological adaptations for reducing urea hydrolysis. The gastrointestinal tract of *Desmodus* is adapted for greater rates of water reabsorption than that of any other mammal (Harlow and Braun 1997), and, after feeding, its kidneys begin instantaneously extracting water from ingested plasma to then be expelled as urine, an ability also believed to help rapidly lower body mass before flight (McFarland and Wimsatt 1969; Singer 2002).

Yet further evidence for adaptive processing of waste products comes from selection on *HOGA1*, which encodes an enzyme responsible for catabolism of hydroxyproline to glyoxylate in liver and kidney mitochondria. Defects in this gene are associated with overproduction of oxalate (Monico et al. 2011), and excess oxalate in the kidneys and urine may result in calcium-oxalate kidney or bladder stones, infection, and organ damage. Furthermore, the glyoxylate cycle, a downstream pathway of beta oxidation during which glyoxylate is combined with acetyl-CoA to produce malate, is thought to be important in the generation of glucose from lipids (Davis et al. 1990). Molecular adaptation in *HOGA1* may thus also present secondary evidence of selection in *Desmodus* for fat-derived energy production. Taken together, our results from a large set of genes offer compelling evidence that the evolution of one of the most unique feeding strategies in all vertebrates has arisen via selection acting on pathways underlying water homeostasis, detoxification, and excretion.

We also found a clear signal of molecular evolution putatively linked to nectarivory, with many PSGs on the Glossophaginae branch known to function in sugar metabolism: *ALDOB*, *MANBA*, *PHKA1*, *PPP1R3A*, *SORD*, and *SLC2A5. ALDOB* encodes a key glycolytic enzyme that reversibly hydrolyzes isomers of phosphorylated fructose and intermediates in glucose metabolism. Fructose is also an important dietary sugar, being a primary monosaccharide in nectar from bat-pollinated flowers (Baker et al. 1998). Independent of insulin, dietary fructose is absorbed and phosphorylated in the liver to fructose-1-phosphate, which, similarly to fructose-6-phosphate in glycolysis, is catabolized by aldolase B. The products of this reaction, glyceraldehyde and dihydroxyacetone phosphate (DHAP), are converted by isomerases to glyceraldehyde-3-phosphate, which may then be converted to phosphoenolpyruvate for the TCA cycle, or, alternatively, used as a substrate for gluconeogenesis to replenish liver glycogen stores. DHAP may also be converted to glycerol for triglyceride synthesis. Selection for efficient glycolysis in nectar-feeding bats might also contribute to their ability to sustain the high metabolic rates needed for hovering flight, a key trait associated with accessing nectar during flower-visiting (Suarez et al. 2011).

*SORD* encodes sorbitol dehydrogenase, which converts sorbitol to fructose in the polyol pathway (Carr and Markham 1995). The synthesis of sorbitol from glucose is considered a strategy for avoiding glucose toxicity. In diabetic patients, unprocessed glucose accumulates in tissues, particularly, the kidneys, eyes, and nerves, and a large percentage is reduced to sorbitol. The resulting osmotic pressure is responsible for many of the tissue damage symptoms of the disease (Burg and Kador 1988). Inferred molecular adaptation in *SORD* in Glossophaginae may thus be a mechanism for avoiding the toxic accumulation of glucose without requiring conventional insulin signaling. Although the functional significance of nonsynonymous substitutions in *ALDOB* and *SORD* remains to be determined, we hypothesize that these changes confer greater efficiency for and/or tighter control over dietary sugar metabolism. Together, these findings constitute the strongest evidence to date of positive selection on glucose and fructose metabolism in nectar-feeding animals and thus have major implications for the understanding of adaptation to high sugar consumption.

Blood sugar levels during feeding and fasting are regulated by glycogenesis and glycogenolysis, and thus, these pathways are likely be especially important for nectar-feeding bats (Qian et al. 2014). Consistent with this, the carbohydrate Reactome PSGs *PHKA1* and *PPP1R3A* both play roles in glycogen metabolism. *PPP1R3A* encodes a subunit of glycogen-associated PP1, and *PHKA1* is a subunit of PHK. Among other roles, PP1 dephosphorylates and inactivates PHK. When active, PHK phosphorylates and activates another enzyme, glycogen phosphorylase, which itself directly catalyzes glycogenolysis. The two antagonistic enzymes PP1 and PHK thus interact crucially in the regulation of glycogen homeostasis. How the detected molecular changes in *PPP1R3A* and *PHKA1* directly affect this critical branch of metabolism is not known; however, mutations in both subunits are linked to serious metabolic disorders. For example, an additional primary role of the PP1 complex is to dephosphorylate GYS, activating it to perform glycogenesis. Knockout mutant mice for the glycogen-targeting *PPP1R3A* subunit showed a 10-fold decrease in skeletal muscle glycogen storage as a result of reduced GYS activity (Delibegovic et al. 2003; Savage et al. 2008), whereas a link between *PPP1R3A* mutations and type 2 diabetes comes from humans (Sánchez-Pozos et al. 2018). Conversely, mutations in *PHKA1* are associated with impaired glycogen breakdown and the inability to supply muscles with glucose (Burwinkel et al. 2003). Our findings add to previous work on bats that uncovered parallel amino acid substitutions in the GYS genes *GYS1* and *GYS2* between frugivorous members of Pteropodidae and Phyllostomidae (Fang et al. 2014; Qian et al. 2014). Although we find no evidence of selection in either GYS gene, our results from glycogen metabolism genes indicate that the evolution of high-sugar diets has involved adaptations in glycogenic pathways.

A final intriguing finding in the nectar bats is positive selection acting on genes that function in fatty acid beta-oxidation, including *SLC22A5* and *CPT1B.SLC22A5* encodes a cation and carnitine transporter (Tamai 2013), which functions in regulating carnitine levels in the liver, kidney, and intestine, whereas *CPT1B* encodes the muscle isozyme of CPT1. Both genes help regulate the transportation of fatty acids to the mitochondria via the carrier protein carnitine (“carnitine shuttle”), where they are oxidized for energy, a process that often occurs when fat reserves are the primary energy source. Thus, these putative molecular adaptations may allow nectar bats to overcome periods of low food availability when glucose reserves are depleted (Voigt and Speakman 2007). It is also noteworthy that, compared with other vertebrates, nectar bats show significantly faster V_max_ capacities for CPT1 and beta-oxidation, hypothesized as a critical requirement for hovering flight (Suarez and Welch 2017).

### A Strong Signal for Craniofacial Evolution

Of the nonmetabolic PSGs identified, many have links to craniofacial development and/or morphology. These PSGs were most abundant on the ancestral branch of the phyllostomid clade, but also occurred across other focal branches, broadly supporting a scenario of historical contingency combined with the emergence of novel adaptations. PSGs at the root of the family include *ADAM28*, *DDHD1*, *GFPT2*, *NUF2*, *OSR1,ADAMTS9*, *HAS2*, *CADM1*, *MAGI2*, and *THSD4*. These loci span roles in tissue architecture and auditory and visual development, with some linked to developmental disorders characterized by distinctive dysmorphisms of stature and face shape (Ludwig et al. 2012; Dard et al. 2017; Cha et al. 2018; Hamer et al. 2018; Holdener et al. 2019). It is thus possible that molecular adaptation in some of these genes provided a backbone for the further craniofacial remodeling for specialized diets, a hallmark of Phyllostomidae (Dumont et al. 2012).

Genes under selection on the Glossophaginae branch included *DDIT4L* and *PRDM16*, both linked to nose morphology, and *HACD4*, *HRH2*, *ISPD*, *SEMA4D*, and *TANC2*, all linked to facial morphology. Similarly, PSGs in *Desmodus* included the morphology-associated genes *ADAMTS8*, *BEST3*, and *CNTNAP2*. It is thus plausible that such loci have contributed to the rostral elongation seen in the Glossophaginae and the rostral shortening/brachycephaly that characterizes the Desmodontinae (Arbour et al. 2019). Other genes potentially important in the evolutionary cranial remodeling of phyllostomids include *PCDH15*, under selection at the origin of plant feeding, and *ABCA4*, *GNB1L*, and *LINGO2*, all under selection in frugivores. In these two branches, the higher numbers of PSGs implicated in craniofacial morphology than in metabolism could arise if development of morphological structures isunder greater evolutionary constraint and thus slower to respond to selection (Smith et al. 1985). Only a few studies to date have examined molecular evolution in candidate developmental genes in plantivorous bats, with one reporting convergent residues in *PAX9* between Neotropical and Old World nectar bats (Phillips et al. 2013). More generally, bats have been proposed as a natural system for the study of facial clefting because some have distinctive facial tissue structuring that resembles developmental defects in humans (Orr et al. 2016). Our finding of selection in a large cohort of loci with links to bone and soft tissue remodeling thus represents a substantial advance, opening up new opportunities for future work.

### Caveats and Conclusions

We find that the extraordinary dietary radiation of phyllostomid bats has been accompanied by positive selection acting on diverse metabolic genes and associated pathways as well as on numerous genes underpinning craniofacial morphology. In most cases, lineage-specific sets of PSGs correlate well with the expected metabolic demands of corresponding diets, implying that PSGs constitute real molecular adaptations. In particular, we find that insectivorous bats show putative molecular adaptations for lipid and protein metabolism, the vampire bat for detoxification and urinary excretion, and nectar-feeders for sugar and glycogen metabolism. Alongside these trends, positive selection was also detected in an unexpectedly broad range of dietary genes in the ancestral phyllostomid, which, coupled with a lack of clear signal at the origin of plant-feeding, suggests historical contingency may underpin the evolutionary shift from animal to plant-based diets.

Although these findings suggest that some molecular changes facilitating dramatic dietary transitions in these bats have occurred in their early evolution, we must also consider whether the different numbers of PSGs detected per lineage might also result from variation in branch lengths in this group. Indeed, given the reference phylogeny (Rojas et al. 2016), the ancestral branch with the most PSGs is also the longest, whereas the ancestral branch of plantivores with fewest PSGs is short. Though longer branches are generally expected to contain more nonsynonymous substitutions than shorter branches, synonymous substitutions also accumulate with branch length; therefore, since all genes were tested with independently optimized trees, the proportion of loci per branch showing ω > 1 will not correlate solely with branch length. In fact, in many evolutionary histories, positive selection occurs episodically and is patchily distributed within a phylogeny (Murrell et al. 2012) such that lineages show acceleration or deceleration of molecular adaptation with respect to the background. In cases where rapid species divergence is associated with the occurrence of adaptive substitutions over short timeframes, insufficient time may pass to allow the accrual of synonymous changes, thereby increasing the chance of detecting positive selection in short branches (dos Reis 2015; Indjeian et al. 2016; Thiltgen et al. 2017). Therefore, given that the numbers of PSGs do not follow this pattern and, in many cases, support our hypotheses based on dietary specialization, it appears that branch length is unlikely to account for the patterns we observe.

Studies that apply selection tests to large cohorts of genes are increasingly common, yet few have sought to examine the underlying distributions of *P* values and their implications for using corrections for multiple tests. Our simulations indicate that the excess of *P* values equal to 1 is likely due to misspecification of the null model in the branch-site test because most proteins are likely to be evolutionarily conserved (Kimura 1983; Eyre-Walker 2006) and will lack a class of sites with ω = 1, as required by the null model. Moreover, we find that the distribution of *P* values < 1 remains nonuniform, implying additional sources of misspecification. Simplistic restrictions on site categorization and ω within the branch-site models may often be violated in real data, whereas the a priori designation of foreground and background branches may not adequately capture the true evolutionary history of a group. Given the null model misspecification in real phylogenomic analysis, further simulation studies are required to understand the statistical behavior of the test under these conditions and obtain guidelines for multiple testing corrections. Until then, it is clear that the branch-site test is conservative and blind application of an inappropriate *P* value correction risks discarding all true positives.

## Materials and Methods

###  

#### Fieldwork

Samples were obtained during fieldwork in Belize (2011), Costa Rica (2013), the Dominican Republic (2014), and Peru (2015) and through donation by the Royal Ontario Museum. In all fieldwork, bats were trapped under permit in high and low mist nets of varying length (4–6 m), generally set up in forest trails or over streams. Individuals of each species, usually adult males, were sacrificed by overdose of isoflurane (Yohe et al. 2019), and multiple tissues were harvested as part of larger projects on genomics and gene expression (Sadier et al. 2018). Tissue was immersed in RNAlater and either flash-frozen in liquid nitrogen in a portable dry-shipper or incubated on ice for around 2 h before being transferred to a freezer.

#### RNAExtraction and Sequencing

To obtain large-scale sequence datasets of protein-coding genes for multiple taxa, we performed RNA-Seq. This approach is more cost-effective than whole genome sequencing (Todd et al. 2016) and has been successfully applied to understand adaptations linked to ecological shifts and new niches in diverse taxa (Davies et al. 2015; Tsagkogeorga et al. 2015; Tong et al. 2017).

RNA was extracted from bat tissue samples with Qiagen RNeasy Mini kits according to standard protocols. RNA was either extracted on a per-tissue basis or as a homogenate of multiple tissues (see supplementary file 1, table S1 for details, Supplementary Material online). cDNA libraries were constructed in-house, using commercially available kits, or by the respective sequencing service. Adapter trimming and phred quality filtering of returned RNA-seq reads was performed with Trimmomatic (Bolger et al. 2014). We used the default 16-base “seed” alignment, with a maximum seed mismatch of two bases, palindromic clipping threshold score of 30, and single-end clipping threshold score of 10. Additionally, a four-base sliding window was employed, with a cut threshold of 15.

#### TranscriptomeAssemblies

Transcriptomes were assembled with Trinity v2.4 (Haas et al. 2013) with default parameters. De novo assemblies for this study were supplemented with RefSeq mRNA transcripts available for seven species from NCBI GenBank and transcriptome assemblies for two bats generated in previous work (Davies et al. 2014). In summary, 100 individual transcriptomic datasets representing 66 bat taxa were generated or collected. For some de novo transcriptomes, tissue-specific RNA-seq datasets from the same individual bat were incorporated in a single Trinity assembly—if generated with the same library preparation method and sequencing platform—whereas other de novo assemblies used only a single RNA-seq dataset (supplementary file 1, Table S1, Supplementary Material online). As tissue collection spanned multiple fieldwork trips over multiple years, some species were harvested more than once from different locations, generating replicate transcriptomes from different individuals either mixed tissue or tissue-specific (supplementary file 1, Table S1, Supplementary Material online). We assessed the presence of mammalian BUSCOs within de novo transcriptomes using BUSCO v4.1.1 in transcriptome mode with database mammalia_odb10 (2020-08-05) (supplementary file 1, Table S2, Supplementary Material online) (Seppey et al. 2019).

#### Ortholog Identification and Alignment

In order to extract orthologous sequences from transcriptomes, a stringent reciprocal BLAST process was used. With blastx, each transcript was queried against a reference database containing the longest protein sequence for each *Homo sapiens* coding gene in the Ensembl human genome release GRCh38.p10 (Release 90, August 2017) (Aken et al. 2017), with an E-value threshold of 1e-06. The reverse tblastn search was also performed. Only the top hit was retained in each case, and only if that top hit was reciprocal in the two searches was the transcript assumed to be homologous to the human gene. The BLAST-aligned portion of the transcript, representing the coding sequence (CDS), was extracted to be used in multiple sequence alignments (MSAs) and all downstream analyses. We also filtered reciprocal blast results to ensure orthology; only genes annotated as Ensembl one-to-one orthologs between human and either of the bat species *Myotis lucifugus* or *Pteropus vampyrus* were retained. In a small number of cases, orthology information for the reference human gene was not annotated in Chiroptera; we retained these. Resultant sequences were filtered for length and premature stop codons using custom Perl scripts.

Using CDSs from collected transcriptome assemblies, an individual MSA was produced per gene. Orthologous CDSs were aligned with PRANK v.170427 (Löytynoja 2014), which provides protein-aware codon-based alignments. Finished alignments were parsed to a single representative sequence per species; the most complete sequence (fewest gaps) was retained. We subsequently removed alignment positions where PRANK had introduced a gap in the reference *Homo sapiens* sequence (i.e. insertions in bats were not considered comparable), and we discarded any sequences comprising more than 50% gaps. Additional filtering masked any positions with missing data from more than 50% of taxa in the alignment. In total, 13,081 orthologous gene alignments were generated.

#### Tests for Molecular Adaptation

To determine the genes that played a role in the dietary radiation of Phyllostomidae, we tested seven branches of the phylogeny for positive selection with maximum likelihood models of molecular evolution. In each case, the branch of interest was labeled as the sole “foreground” branch, and the rest of the tree was left unlabeled as the “background.” The seven branches of interest were the ancestral branch of Mormoopidae (control branch), 1) the ancestral branch of Phyllostomidae, 2) the branch of the common vampire bat (*Desmodus rotundus)*, 3) the ancestral branch of an insectivorous subfamily Phyllostominae, 4) the ancestral branch of the plantivorous clade, 5) the ancestral branch of a nectarivorous subfamily Glossophaginae, and 6) the ancestral branch of the frugivorous clade comprising subfamilies Carolliinae, Rhinophyllinae, and Stenodermatinae (fig. 1). Note, we use plantivory as a blanket term for any plant-derived nutrition, e.g. nectarivory and frugivory.

We performed tests for molecular adaptation using codeml in PAML v4.9e (Yang 2007). Given a species tree and MSA, codeml uses maximum likelihood models of molecular adaptation to estimate synonymous and nonsynonymous substitution rates for the given data. The nonsynonymous/synonymous substitution rate ratio, d_N_/d_S_ or ω, exceeds one in the case of positive selection, is approximately equal to one when evolving neutrally, and is less than one where purifying selection favors conservation of protein sequence. An estimate of ω can be obtained for either sites (codons) in a gene, branches of a phylogeny, or particular sites on particular branches under different maximum likelihood models (Yang 2014).

To test for inferred molecular adaptation due to positive selection, we implemented the improved branch-site model A with codeml (Yang and Nielsen 2002; Zhang et al. 2005). Specifically, Test 2 of the branch-site Model A was used as the test for selection and compared with null Model A to assess whether positive selection is a better fit than neutral evolution. Each model performs site-wise estimation of ω for the branch of interest (foreground) and other branches (background). Model A assigns sites to one of four constrained ω site categories: ω_0 _< 1 (purifying selection); ω_1_ = 1 (neutral selection); ω_2a _> 1 (positive selection) in foreground, purifying in background; and ω_2b _> 1 in foreground, neutral in background. The null model for Model A differs in that the value of ω in site class 2 cannot exceed 1 in the foreground branch. The strength of fit of Model A versus the null model was assessed using the LRT, in which significance is determined by a χ^2^ test with 1 degree of freedom.

For each of the seven lineages in turn, the Model A test for positive selection and corresponding null model were run in codeml individually for each gene alignment, using Queen Mary’s Apocrita HPC facility, supported by QMUL Research-IT (King et al. 2017). Prior to launching analyses, alignments were checked for appropriate taxonomic representation; given a branch of interest, the gene was only tested if the alignment contained at least two descendants of said branch (except in the case of the vampire bat, *Desmodus rotundus*). Additionally, all alignments with fewer than 10 total taxa were omitted. The tree topology used for selection tests followed Rojas et al. (2016), unrooted, and trimmed to match alignment taxon representation using Newick Utilities v1.6 (Junier and Zdobnov 2010) (fig. 1). Branch-lengths were optimized in codeml during model fitting. codeml implements BEB estimation of the posterior probabilities that sites belong to assigned classes (Yang et al. 2005). Resulting model likelihoods, for Model A and the null model, and BEB values were parsed. LRTs were performed in R with the function chitest.

#### Simulations to Examine Branch-Site Model Misspecification

In high-throughput omics analyses, authors typically apply *P* value corrections, such as Benjamini–Hochberg FDR, to account for multiple testing and the possibility of false positives. This approach assumes that *P* values are uniformly distributed when the null model is true (Strimmer 2008; Noble 2009), although this is rarely tested. Therefore, we first plotted the frequency of empirical *P* values from our large cohort of gene-wise branch-site LRTs and found a highly nonuniform distribution (see Results section). Nonuniformity of *P* values is commonly caused by null model misspecification (Pounds and Cheng 2005; Strimmer 2008), which appears not to have been explored previously in the context of branch-site phylogenomic analyses.

To examine this in greater detail, we used a simulation approach detailed below. The factors we needed to consider were as follows. In the alternative model of the branch-site test of positive selection, a proportion of codon sites, $p_{2}$, is assumed to evolve under positive selection with $\omega_{2}\geq 1$ in the foreground branches of the phylogeny. In the null model, the same fraction of codons in the foreground branches evolves neutrally (i.e. $\omega_{2}=1$). Thus, in the null model, $\omega_{2}=1$ is at the boundary of the parameter space because $\omega_{2}<1$ is forbidden in the alternative model. Under such boundary conditions, the asymptotic distribution of the log-likelihood-ratio statistic is a 50:50 mixture of a $\chi^{2}$ distribution and point mass 0 (Self and Liang 1987), which has been confirmed for the branch-site test by computer simulations (Yang and dos Reis 2011). Thus, *P* values obtained using the $\chi^{2}$ distribution are ordinarily divided by two to correct for the boundary condition, prior to any post-hoc corrections to account for multiple tests.

However, many real proteins are highly conserved with average ω≪1 across sites and with no proportion of sites evolving neutrally. Thus, the null model will be misspecified because the true value of the ω parameter is not at the boundary of the parameter space but rather deep inside in the forbidden zone of the alternative model (see supplementary fig. 1 in SI, Supplementary Material online). For these proteins, the log-likelihood-ratio statistic will have a much larger proportion of values at point mass zero than if the null model was not misspecified. If this large proportion at mass zero in the likelihood-ratio is not taken into account, any post-hoc correction applied to the entire set of *P* values, such as FDR, will be too conservative and fail to detect true positives.

Therefore, to illustrate this problem, we carried out a set of simulations under boundary conditions with and without null model misspecification. First, we simulated 10,000 samples of size 100 from a normal distribution with μ=0 and σ=1, and carried out a likelihood-ratio test of the hypothesis H0: μ=0 vs. H1: μ>0, that is, under a boundary condition. Second, we repeated the simulation above but with 1,000 samples from the alternative model (i.e., with μ>0), such that, for 10% of cases, the null model is false. Finally, we repeated the previous simulation, but this time, 4,500 samples were obtained from a misspecified null model (^i.e., with μ<0), with a further 4,500 samples from the correct null model and 1,000 from the alternative model.

#### Results Filtering and Robust FDR

Based on simulations and the observed frequency of *P* values from our selection tests, we chose to consider genes at the standard 5% threshold without FDR as putative cases of positive selection, whereas also keeping the test conservative by not halving *P* valuesand by applying additional post-hoc filtering. First, we ensured that each gene for which the LRT was significant had at least one site identified by BEB as belonging to site Class 2a or 2b with a posterior probability > 0.5. Genes without BEB sites were discounted. Second, if the gene had > 5 such sites, it was only retained if the median interval between BEB sites was 10 or more amino acids. Genes with a median interval < 10 were discounted. This secondary filtering step helps reduce false positives due to alignment error, where a large concentration of BEB sites may be identified at poorly aligned regions (Tsagkogeorga et al. 2015; Davies et al. 2018). For all lineages tested, this filtering process reduced the number of retained significant results by between 17.5% and 60.9% per lineage and 28.1% overall. We report these post-filtering significant results as PSGs.

From the empirical *P* value distribution, we diagnosed those *P* values that are likely to be artifacts of model misspecification (>0.98). Among our PSGs, we highlighted loci with greatest significance by using the robust-FDR method of Pounds & Cheng (2006) on the pooled cohort of *P* values < 0.98 from all branches. This method does not assume that *p* is derived from a two-sided test and thus performs more favorably when a poorly specified one-sided null model produces an excess of *P* values at the right tail.

#### Functional Roles of PSGs

Following selection tests, PSGs were cross-referenced against metabolic “Reactomes,” which describe pathways for processing dietary macro and micronutrients (Croft et al. 2014; Fabregat et al. 2018). We were predominantly interested in three major Reactomes: the metabolism of amino acids and derivates, the metabolism of lipids, and the metabolism of carbohydrates. We also considered four additional minor Reactomes: digestion and absorption, metabolism of vitamins and cofactors, integration of energy metabolism, and the citric acid cycle and respiratory electron transport (categories are not mutually exclusive).

In addition, we independently examined links between PSGs and phenotypic traits determined by genome-wide association studies (GWAS), particularly traits pertaining to craniofacial morphology or kidney function/kidney disease (see SI for a list of selected traits). After thus establishing a possible role in dietary metabolism, morphology, or urinary excretion, the biological relevance of PSGs to our study system was inferred from additional GWAS associations, such as metabolic disease, and literature searches.

#### Functional Enrichment of PSGs

For each lineage, ontology enrichment in PSGs was tested with topGO in R using the “weight01” algorithm and Fisher’s exact test for significance (*P *<* *0.05) (Alexa and Rahnenfuhrer 2016). BP GO term annotations were taken from Ensembl. The background for the enrichment test used in each case was the full cohort of genes tested for the specific lineage. The topGO procedure takes the hierarchical structure of GO terms into account such that multiple testing of terms is nonindependent, and thus resulting *P* values were not corrected per author recommendations, although we restrict discussion to *P *<* *0.01.

## Supplementary Material

Supplementary data are available at *Molecular Biology and Evolution* online.

## Supplementary Material

msab028_Supplementary_Data
