# Erratum to: Dietary Diversification and Specialization in Neotropical Bats Facilitated by Early Molecular Evolution

**DOI:** 10.1093/molbev/msab204

**Published:** 2021-08-18

**Authors:** Joshua H T Potter, Kalina T J Davies, Laurel R Yohe, Miluska K R Sanchez, Edgardo M Rengifo, Monika Struebig, Kim Warren, Georgia Tsagkogeorga, Burton K Lim, Mario dos Reis, Liliana M Dávalos, Stephen J Rossiter

*Molecular Biology and Evolution*, msab028, https://doi.org/10.1093/molbev/msab028

When the pdf of this paper was first published, the figures were missing and supplementary data from the wrong paper was uploaded. This has now been corrected online. The publisher apologises for this error.

