# Supplementary material for: Dietary Diversification and Specialization in Neotropical Bats Facilitated by Early Molecular Evolution
Source: Mol Biol Evol. 2021 Mar 4;38(9):3864–83. doi: 10.1093/molbev/msab028 (PMC8382914; doi:10.1093/molbev/msab028)
Supplement: msab028_Supplementary_Data [file msab028_supplementary_data.zip › Supplementary Info - Suppl Results and Methods.docx]

Supplementary Information for:

***Dietary diversification and specialisation in Neotropical bats facilitated by early molecular evolution***

Potter *et al.*, 2020

**Supplementary Results**


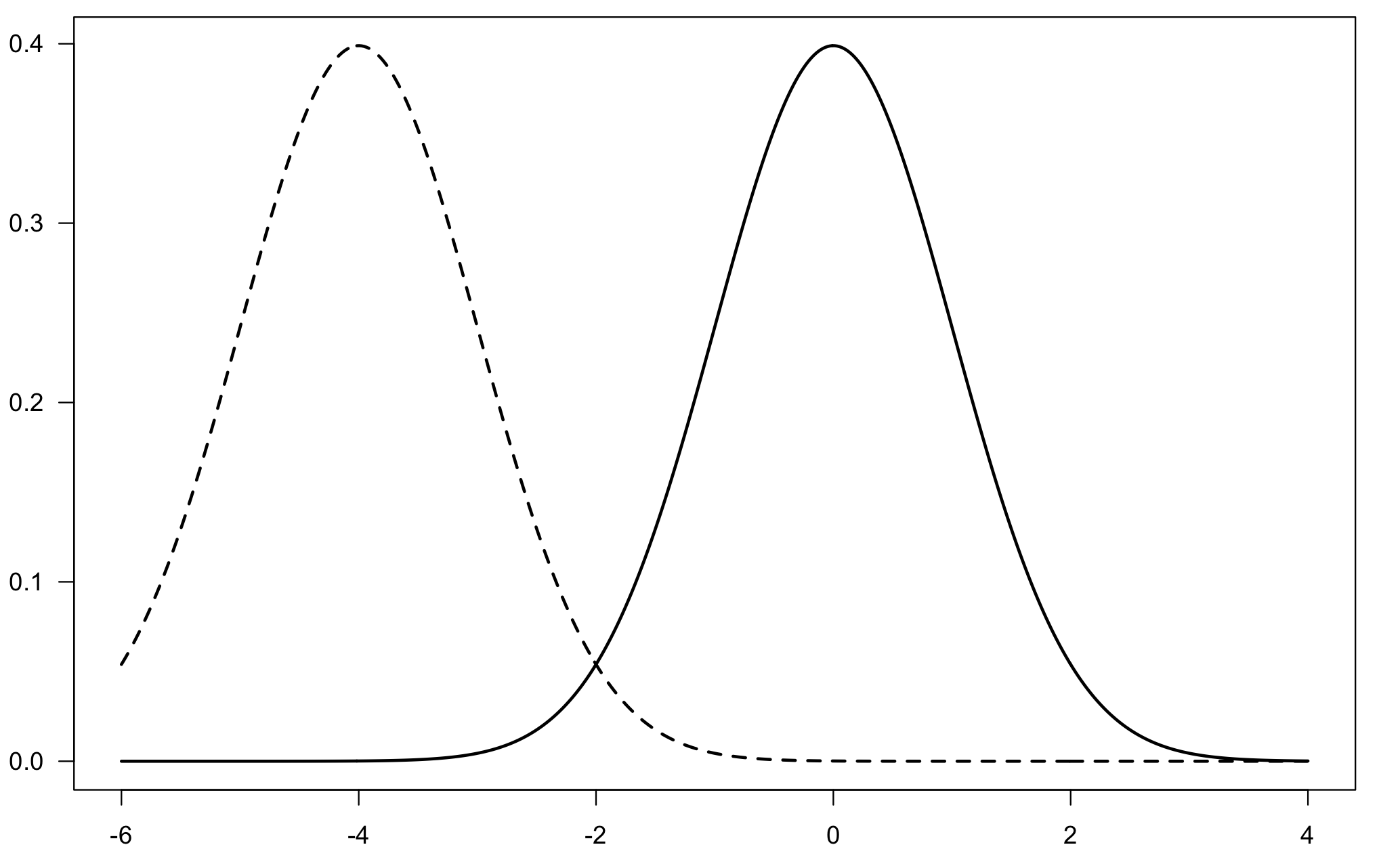


Forbidden zone: Parameter estimates here (i.e. ω < 0) are not allowed.

Boundary condition:

H0: ω = 0

H1: ω > 0

Mis-specified null hypothesis: the true parameter value is deep inside the forbidden zone.

ω

Probability density

This curve gives the distribution of the estimate of ω given the true value ω = 0.

**Supplementary Fig. 1:** **Null model misspecification**

Null model restrictions on ω, which produce model misspecification.


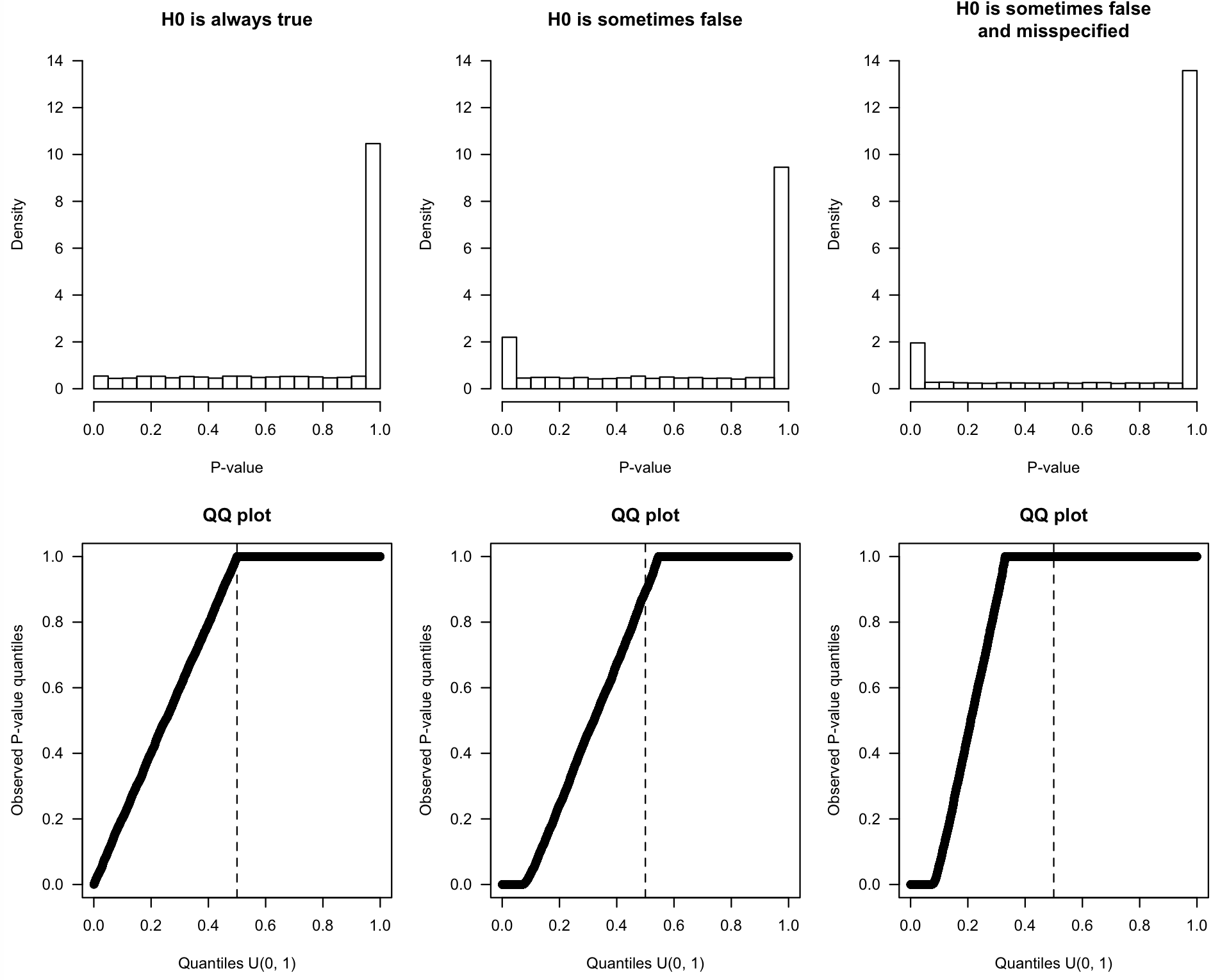


**Supplementary Fig. 2: p-value distributions from simulations**

From left to right: A) null hypothesis is always true. B) 10% true positives. C) null model misspecification

**Supplementary Methods**

*Craniofacial GWAS traits:*

cleft palate, optic cup area, chin dimples, facial wrinkles, craniofacial microsomia, facial morphology (factor 23), facial morphology, permanent tooth development, axial length, intracranial volume, facial morphology (factor 10, width of nasal floor), orofacial clefts, facial morphology (factor 19), eye morphology, graves' disease, facial morphology (factor 14, intercanthal width), facial morphology (factor 20), age-related cataracts, nose morphology, nose size, maximum cranial width, vertical cup-disc ratio, facial morphology (factor 11, projection of the nose), facial morphology (factor 7, width of cartilaginous portion of nose), optic disc area, facial morphology (factor 13, vertical position of alar curvature relative to upper lip), corneal curvature, cleft lip with or without cleft palate, nonsyndromic cleft lip with or without cleft palate, bone mineral density (paediatric, skull), facial morphology (factor 1, breadth of lateral portion of upper face), facial morphology (factor 3, length of philtrum), erosive tooth wear (severe vs none or mild), nonsyndromic cleft lip with cleft palate, lobe attachment (rater-scored or self-reported), lobe attachment (rater scored)

*Kidney function/disease GWAS traits:*

estimated glomerular filtration rate, serum uric acid levels, blood urea nitrogen levels, glomerular filtration rate, chronic kidney disease, glomerular filtration rate (creatinine), serum metabolite concentrations in chronic kidney disease, urate levels, gout, proteinuria and chronic kidney disease, serum metabolite ratios in chronic kidney disease, renal function-related traits (urea), hyperuricemia, renal overload gout, uric acid levels, gout vs. hyperuricemia, serum urea levels, gallstone disease, urate levels in lean individuals, urinary metabolites (h-nmr features), estimated glomerular filtration rate in non-diabetics, urinary sodium excretion, membranous nephropathy, mild to moderate chronic kidney disease, diabetic kidney disease, chronic kidney disease (severe chronic kidney disease vs normal kidney function) in type 1 diabetes, kidney function decline traits, urinary albumin excretion (no hypertensive medication), urinary albumin excretion, total bilirubin levels, glucosuria (moderate to severe), glucosuria, renal underexcretion gout, urate levels in overweight individuals, kidney stones, urinary albumin-to-creatinine ratio in non-diabetics, urate levels (bmi interaction), gout in chronic kidney disease, serum urate levels in chronic kidney disease, urinary metabolites, urinary sodium to creatinine ratio, urinary potassium to creatinine ratio, glomerular filtration rate in non diabetics (creatinine)
